# Matrix metalloproteinase 9 expression and glioblastoma survival prediction using machine learning on digital pathological images

**DOI:** 10.1038/s41598-024-66105-x

**Published:** 2024-07-02

**Authors:** Zijun Wu, Yuan Yang, Maojuan Chen, Yunfei Zha

**Affiliations:** Department of Radiology, Renmin Hospital of Wuhan University, Wuhan, 430000 China

**Keywords:** Glioblastoma, Pathological image, MMP9, Machine learning, Classification and prognosis prediction, Prognostic markers, Molecular medicine, CNS cancer, Oncogenes, Tumour biomarkers, Prognostic markers, Bioinformatics, Gene expression analysis, Molecular imaging, Image processing, Machine learning, Predictive medicine

## Abstract

This study aimed to apply pathomics to predict Matrix metalloproteinase 9 (MMP9) expression in glioblastoma (GBM) and investigate the underlying molecular mechanisms associated with pathomics. Here, we included 127 GBM patients, 78 of whom were randomly allocated to the training and test cohorts for pathomics modeling. The prognostic significance of MMP9 was assessed using Kaplan–Meier and Cox regression analyses. PyRadiomics was used to extract the features of H&E-stained whole slide images. Feature selection was performed using the maximum relevance and minimum redundancy (mRMR) and recursive feature elimination (RFE) algorithms. Prediction models were created using support vector machines (SVM) and logistic regression (LR). The performance was assessed using ROC analysis, calibration curve assessment, and decision curve analysis. MMP9 expression was elevated in patients with GBM. This was an independent prognostic factor for GBM. Six features were selected for the pathomics model. The area under the curves (AUCs) of the training and test subsets were 0.828 and 0.808, respectively, for the SVM model and 0.778 and 0.754, respectively, for the LR model. The C-index and calibration plots exhibited effective estimation abilities. The pathomics score calculated using the SVM model was highly correlated with overall survival time. These findings indicate that MMP9 plays a crucial role in GBM development and prognosis. Our pathomics model demonstrated high efficacy for predicting MMP9 expression levels and prognosis of patients with GBM.

## Introduction

Glioblastoma (GBM), classified as a World Health Organization (WHO) grade 4, is the prevailing primary malignant brain cancer that originates predominantly from neural stem or progenitor glial cells. Despite aggressive surgical intervention followed by adjuvant postoperative radiotherapy and chemotherapy, the five-year survival rate for individuals diagnosed with GBM remains less than 10%^1^. The hallmark of GBM is its prominent genetic heterogeneity, necessitating precise genetic data for accurate diagnosis and prognostic assessment. Certain biomarkers, such as IDH mutation and 1p/19q codeletion status, have emerged as clinically relevant prognostic factors. However, traditional biomarkers are no longer adequate for the demands of personalized precision medicine.

The tumor microenvironment has an essential function in determining the prognosis of glioblastoma^2,3^. Matrix metalloproteinase 9 (MMP9) contributes to the tumor microenvironment^4^. MMP9 encodes matrix metalloproteinase 9, a member of the matrix metalloproteinase (MMP) family. The MMP family of proteins participates in normal physiological processes, such as reproduction, embryonic development, and tissue remodeling, as well as pathological processes involving extracellular matrix degradation, such as in arthritis and metastasis^5^. Andecaliximab (ADX), a monoclonal antibody, suppresses the extracellular enzyme MMP9, which has an essential function in matrix remodeling, tumor development, and metastasis^6^. Phase I and phase Ib clinical trials observed promising efficacy in individuals with gastric or gastroesophageal junction adenocarcinoma when treated with a combination of modified oxaliplatin, leucovorin, and fluorouracil (mFOLFOX6) along with ADX. The median progression-free survival was 7.8 months among all patients and 9.9 months among those who received first-line treatment, while the overall response rates were 48% and 50%, respectively^6^. These studies suggest targeting MMP9 as a promising therapeutic strategy for glioblastoma.

However, the current methods for MMP9 expression detection present several limitations, including 1) difficulty in collecting fresh tissue samples for mRNA or protein level detection, which is also operator- and antibody-dependent; 2) limitations of paraffin-embedded tissue sample-based detection due to operator and antibody effects; and 3) high cost. Therefore, efficient methods are required to assess MMP9 expression in GBM.

Hematoxylin and eosin (H&E)-stained slides are indispensable for clinical diagnosis and provide the most readily available image data. Pathomics involves converting pathological images into high-throughput data using artificial intelligence (AI), encompassing quantitative features including morphology, texture, and biology, to facilitate the pathological diagnosis and evaluation of molecular expression levels. The gradual integration of AI in pathology is bringing about significant changes in decision-making processes, specifically in oncology. Benoît and coworkers have pursued an AI model integrating multiple data to predict RNA sequencing (RNA-Seq) expression in tumors using H&E histology^7^. A recent deep learning model based on H&E-stained images predicted microsatellite instability, a genetic biomarker in multiple cancers, including colorectal cancer^8^. Another study predicted BRCA1-associated protein 1 expression in uveal melanoma^9^. However, MMP9 expression levels in GBM have not yet been assessed in pathomics studies.

Herein, we employed The Cancer Genome Atlas (TCGA) and Cancer Imaging Archive (TCIA) databases to construct a pathomics model that integrates H&E-stained whole slide images (WSIs) with machine learning techniques to evaluate the MMP9 expression level in GBM and explore its underlying molecular pathological characteristics.

## Material and methods

### Data source

**Figure 1:**
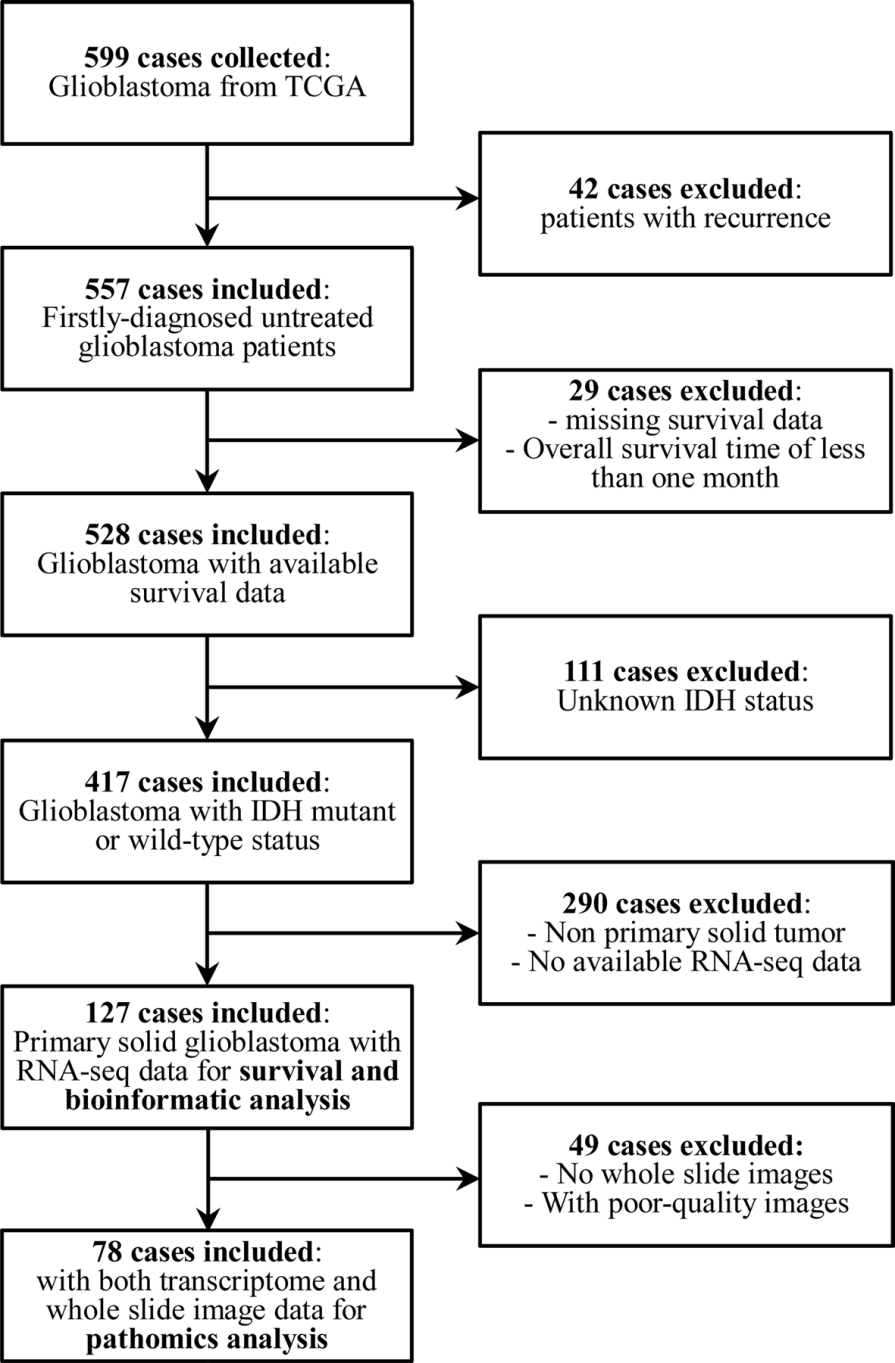
Flowchart of patient inclusion and exclusion in this retrospective study.

Digitized records of RNA-Seq data and pathological images of 599 patients with GBM were obtained from TCGA (https://www.cancer.gov/ccg/research/genome-sequencing/tcga) and TCIA databases (https://dev.cancerimagingarchive.net/). The 5 cases of adjacent non-tumor normal tissues from TCGA and 1152 cases of non-lesion normal tissues from Genotype-Tissue Expression (GTEx) database were included to calculate the expression of MMP9 in normal tissues. After excluding patients with recurrence, those with missing survival data, an overall survival (OS) time of less than one month, patients without IDH mutation status, and patients without RNA-seq data, 127 cases were included in the bioinformatics analysis. In the pathomics analysis, we included 78 patients with both transcriptome and WSI data, and excluded patients with poor-quality WSIs (Fig. 1). The WSIs were formalin-fixed and paraffin-embedded tissue sections in svs format with a maximum magnification of 20 × or 40 × ^10,11^. Among the 78 samples, there are 46 samples at 20X and 32 samples at 40X magnification. Toil software processed the raw data from RNA-Seq to obtain transcripts per kilobase (TPM) values for normalization^12^. The clinical data included age, sex, tumor grade, treatment protocol (radiotherapy or chemoradiotherapy), and the statuses of MGMT promoter and IDH. Patients with mutations in the IDH1 and IDH2 genes are defined as IDH mutant, while others are defined as IDH wild-type. Based on the DNA methylation of the MGMT gene promoter, patients are categorized as unmethylated, methylated, or unknown. Patients are defined as yes or no based on whether they received chemotherapy and radiotherapy.

### TCGA cohort-based prognostic analyses

To categorize the GBM cohort into high- and low-MMP9 groups, we used R package survminer based on minimum p-value method. We obtained MMP9 RNA-seq data in log2(FPKM + 1) format, and determined the optimal cutoff value for MMP9 in survival analysis using the "surv_cutpoint" function in survminer package. The survival package in R was used to assess the prognostic significance of MMP9 and clinicopathological characteristics for GBM. Kaplan–Meier curves were initially employed to analyze survival for each variable, and the disparities in survival probability among different groups were assessed using the log-rank test. A univariate Cox proportional hazards regression analysis was conducted to identify the variables significantly related to OS. A multivariate Cox regression analysis was performed to determine the independence of these significant variables from one another. Subsequently, subgroup analysis was performed using univariate Cox regression to assess MMP9 impact on survival in various clinical subgroups. An interaction term was included in the Cox model to examine the interaction between MMP9 and the subgroup variable for OS within each subgroup.

### Pathological image processing and feature extraction

**Figure 2:**
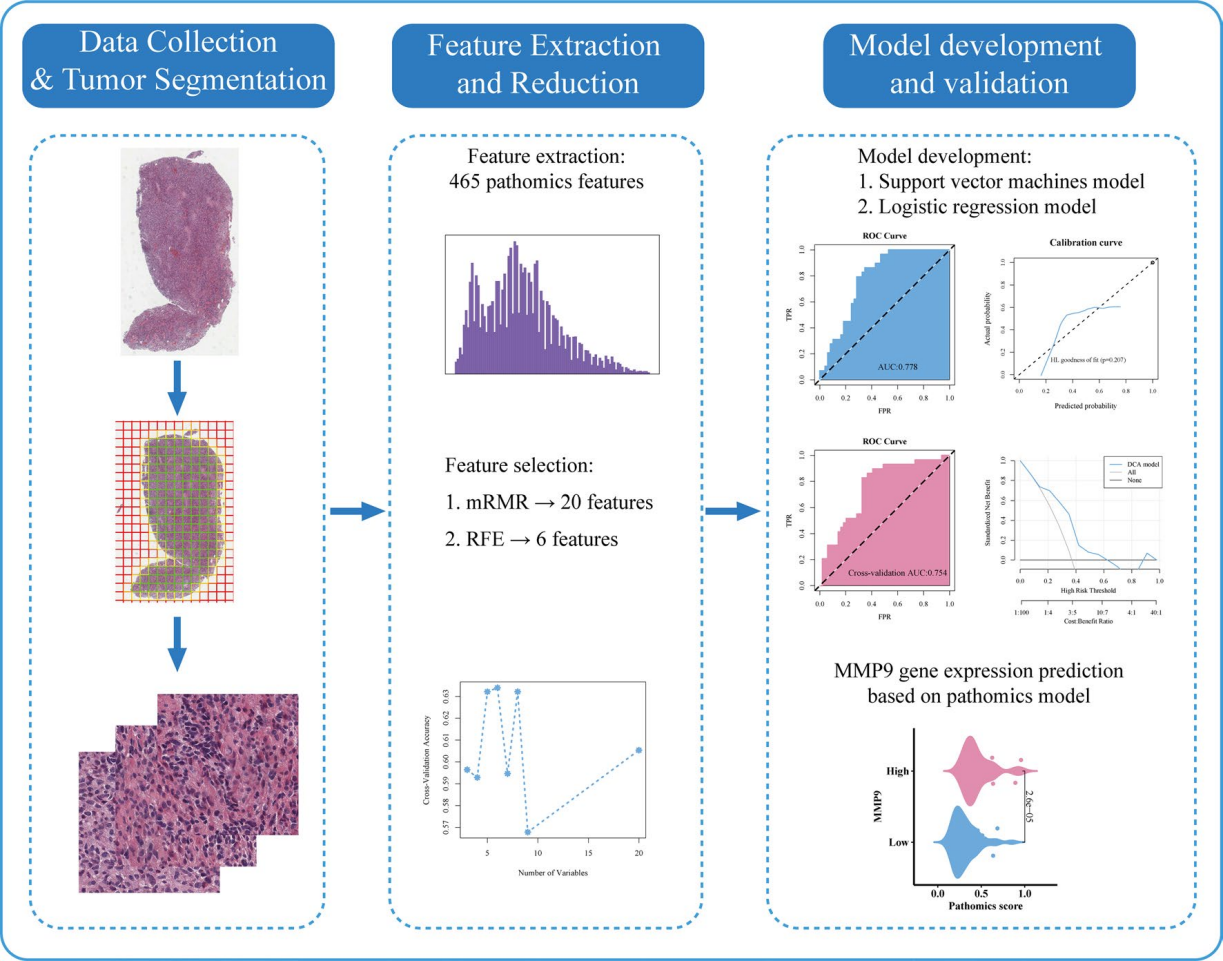
Overall workflow and pipeline of the study.

The pathomics-processing pipeline is illustrated in Fig. 2. We performed image segmentation using the OTSU algorithm in the Openslide Python library^13^. The OTSU algorithm is a type of image binarization segmentation threshold algorithm that uses the threshold to automatically segment the image into two sections: an undesirable background and the tissue area needed for research. The 40 × image was segmented into multiple 1024 × 1024 pixel subimages. Similarly, the 20 × image was segmented into multiple 512 × 512 pixel subimages, which were subsequently upsampled to a resolution of 1024 × 1024 pixels. Next, subimages with white space greater than 50% were excluded. For each patient, ten subimages were randomly chosen from each WSI for subsequent analysis ^10,11^.

A total of 465 image features were extracted from each image tile using texture analysis via PyRadiomics (version 3.0, https://pyradiomics.readthedocs.io/en/latest/ (accessed on November 26, 2022). These features included morphological, first-, second-, and high-order features. The mean value of each extracted feature from the chosen ten tiles was summarized to denote the value of the corresponding slide for further statistical analysis. During preprocessing, the image features were standardized and selected based on the features in the image. Using the z-scores, image features were standardized using the average and standard deviation for each feature.

### Feature selection

To eliminate potential redundancy and prevent overfitting, we used the maximum relevance and minimum redundancy (mRMR) algorithm along with the recursive feature elimination (RFE) algorithm for feature screening. The mRMR approach, implemented using the mRMR package available in R CRAN, was deployed to rank the significance of features by maximizing the measure of mutual information (MI) between class labels while simultaneously minimizing the MI between the remaining features. Subsequently, the mRMR-selected features were further screened using the RFE, gradually reducing their number. The prediction model was trained on the original features with assigned weights. A recursive procedure was then applied to eliminate features with minimal absolute weights until the desired number was reached.

### Logistic regression (LR) and support vector machine (SVM) model establishment

Two machine learning approaches were used to develop the pathomics prediction models: LR and SVM. The remaining features with nonzero coefficients were selected and incorporated into a multivariate backward stepwise LR, with the minimal Akaike Information Criterion (AIC) used to establish the model. The selection of the SVM as a classifier was primarily driven by its capacity to generate classification hyperplanes with narrow margins between the hyperplane and the nearest instances. We selected the best-performing model for adoption and calculated the pathomics score (PS). The SVM model outputs the probability of forecasting *MMP9* expression levels. The Wilcoxon test was used to compare the disparities in PS between the high and low *MMP9* expression groups.

### Model validation and evaluation

We used ROC analysis to estimate the diagnostic significance of the existing models. The evaluation metrics encompassed accuracy (ACC), specificity (SPE), sensitivity (SEN), positive predictive value (PPV), and negative predictive value (NPV). Pathomics model calibration was determined using a calibration curve and the Hosmer–Lemeshow goodness-of-fit test. The overall performance of the model was evaluated using the Brier score, a measure package in R. To calculate the area under the ROC curve, we used the pROC package in R. Decision curve analysis (DCA) was performed using the rmda package in R.

### Pathomics score related gene enrichment analysis

Gene Set Enrichment Analysis (GSEA) was performed using the ClusterProfiler package in R, which revealed various enriched pathways. Subsequently, the GSEA results were obtained by ranking all known genes based on their enrichment scores, ranging from the most positive to the most negative. A total of 1000 random sample permutations were performed. GSEA was performed using Kyoto Encyclopedia of Genes and Genomes (KEGG, c2.cp.kegg.v7.5.1. symbols.gmt)^14,15^ and Hallmark (h.all.v7.5.1. symbols.gmt) gene set collections. A *p-*value of < 0.05 and False discovery rate of < 0.25 were considered significant. Spearman's ρ rank test assessed correlations between PS and angiogenesis-related genes^16^ and apoptosis-related genes^17^. In the analysis of immune infiltration, the gene expression profiles (in TPM format) were imported into CIBERSORTx (https://cibersortx.stanford.edu/) to analyze the relative fractions of 22 types of immune cells using 1000 permutations^18^. Subsequently, we compared the variations in immune cell fractions between high- and low-PS groups using the R package limma.

### Statistical analysis

The Wilcoxon rank-sum test was used to analyze differences in MMP9 expression levels between GBM and normal tissues. The Wilcoxon rank-sum test was employed to compare continuous clinicopathological variables, while Pearson's χ2 test was used to compare categorical variables. Hazard ratios (HR), 95% confidence intervals (CI), and *p*-values were calculated to present the results of the Cox regression model. Pathological feature extraction and selection were conducted using Python 3.6.0, and all statistical analyses were conducted using R version 3.6.2. The two-tailed significance level was set at *p* < 0.05.

## Results

### Prognostic value of MMP9

**Figure 3:**
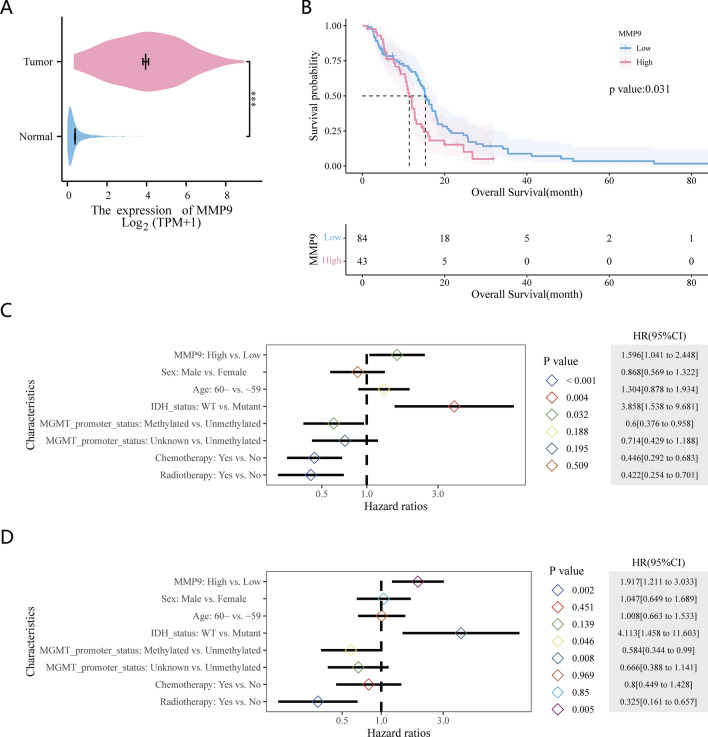
The expression and prognostic significance of MMP9 in glioblastoma (GBM) according to TCGA. (**A**) Higher MMP9 expression in GBM compared with normal tissues. (**B**) Kaplan–Meier curve showing the correlation between elevated MMP9 expression with a bad overall survival. (**C**) Univariate and multivariate Cox regression analysis of the prognostic value of MMP9 and clinicopathologic factors.

As shown in Fig. 3A, MMP9 expression was significantly higher in GBM tissues than in normal brain tissues. Figure 3B shows the Kaplan–Meier curves, which demonstrate a significant link between elevated MMP9 expression and poor OS (*p* = 0.031). The median survival time of the group with low and high MMP9 expression was 15.3 and 11.4 months, respectively. Univariate Cox regression analysis for OS in Fig. 3C illustrates that upregulated MMP9 correlated with poor survival (HR 1.596, 95% CI 1.041–2.448, *p* = 0.032). In multivariate Cox regression analysis (Fig. 3D), MMP9 showed an independent predictive capacity for OS (HR 1.917, 95% CI 1.211–3.033, *p* = 0.005), as did IDH status, MGMT promoter status, and radiotherapy, whereas age, sex, and chemotherapy did not.

### Patient characteristics

Data from 127 patients were included in this study. Patients were divided into a high MMP9 expression group (n = 43) and a low expression group (n = 84) according to MMP9 expression, with 4.2784 as the cutoff value. The patients’ clinical information is shown in Table 1. Patients from the training and test datasets had similar sex distribution, age, and IDH, MGMT promoter, chemotherapy, and radiotherapy statuses (*p* > 0.05).

**Table 1:** Clinical characteristics of patients with glioma from TCGA.

Variables	Total (n = 127)	Low MMP9 (n = 84)	High MMP9 (n = 43)	*p*
Sex, n (%)				0.878
Female	41 (32)	28 (33)	13 (30)	
Male	86 (68)	56 (67)	30 (70)	
Age, n (%)				0.581
≤ 59	56 (44)	39 (46)	17 (40)	
60 ≥	71 (56)	45 (54)	26 (60)	
IDH_status, n (%)				0.271
Mutant	9 (7)	8 (10)	1 (2)	
WT	118 (93)	76 (90)	42 (98)	
MGMT_promoter_status, n (%)				0.421
Unmethylated	59 (46)	41 (49)	18 (42)	
Methylated	41 (32)	28 (33)	13 (30)	
Unknown	27 (21)	15 (18)	12 (28)	
Chemotherapy, n (%)				0.897
No	36 (28)	23 (27)	13 (30)	
Yes	91 (72)	61 (73)	30 (70)	
Radiotherapy, n (%)				0.513
No	20 (16)	15 (18)	5 (12)	
Yes	107 (84)	69 (82)	38 (88)	

### LR and SVM model performance

**Figure 4:**
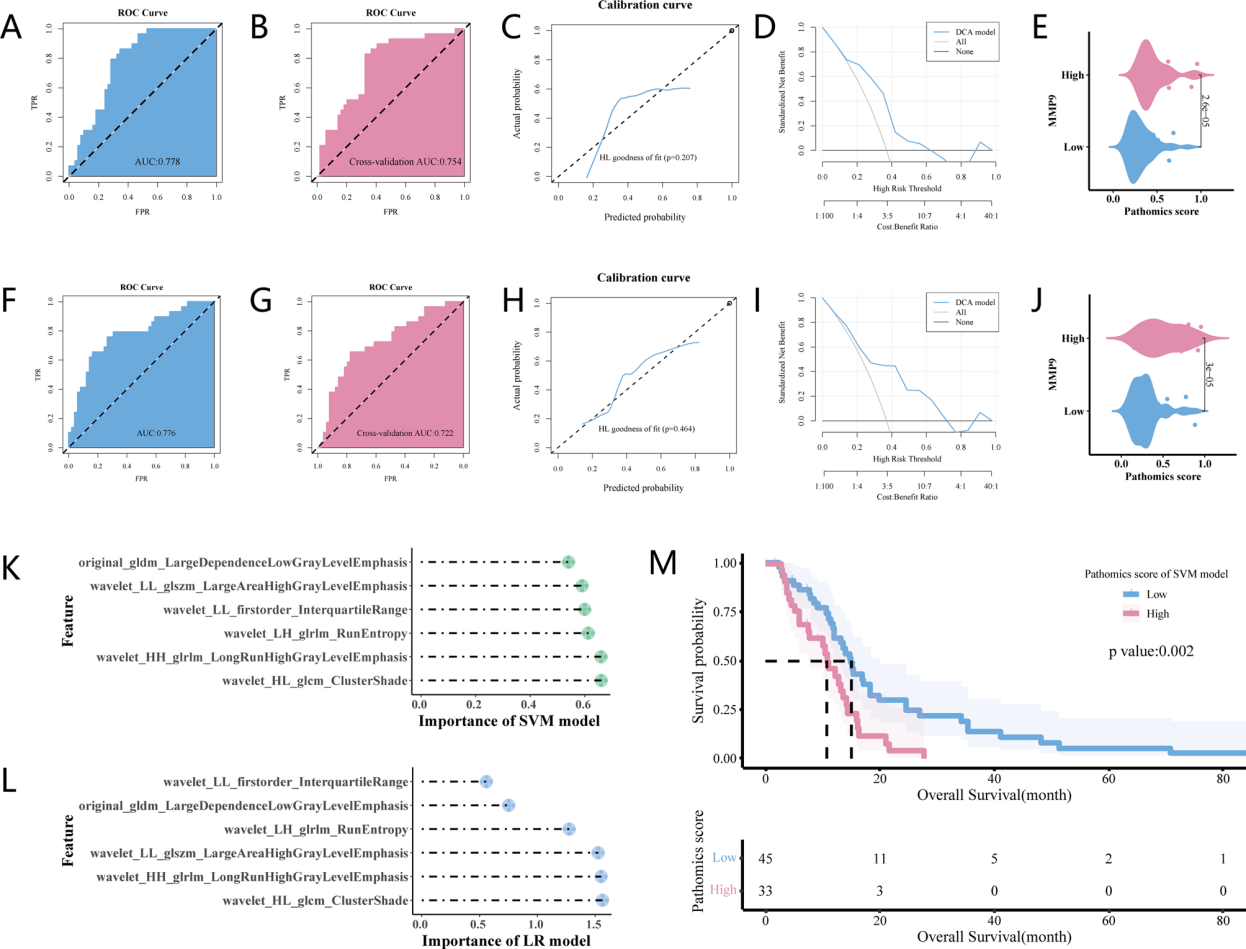
Pathomics models for predicting MMP9 expression and survival in patients from paired TCIA and TCGA GBM dataset. (**A**, **B**) The support vector machine (SVM) model ROC curves in the training and validation sets, with (**C**, **D**) the corresponding calibration curve and decision curve analysis (DCA), and (**E**) pathomics score (PS) between patients with high and low MMP9 expression. (**F**, **G**) ROC curves of the logistic regression (LR) model in the training and validation sets, with (**H**, **I**) the relevant calibration curve and DCA, and (**J**) PS between patients with high and low MMP9 expression. (**K**, **L**) The importance of the six optimal features to SVM and LR models, respectively. (**M**) PS association with overall survival in a Kaplan–Meier curve.

Figure 4 depicts the MMP9 gene expression prediction performance in both the training and validation cohorts. The SVM model demonstrated strong predictive capability, with an AUC of 0.778 (95% CI 0.677–0.879, Fig. 4A), whereas tenfold cross-validation yielded an AUC of 0.754 (95% CI 0.644–0.865, Fig. 4B). The calibration curve and Hosmer–Lemeshow goodness-of-fit test indicated a high level of agreement between the SVM model's predictions and actual observations (*p* > 0.05, Fig. 4C). Furthermore, the DCA confirmed the clinical practicality of the SVM model (Fig. 4D). Notably, within the SVM model, patients with high PS exhibited elevated MMP9 expression levels (Fig. 4E). The LR model also established a favorable predictive capability, as evidenced by an AUC of 0.776 (95% CI 0.665–0.886) in Fig. 4F, whereas cross-validation yielded an AUC of 0.722 (95% CI 0.604–0.841, Fig. 4G). The calibration curve indicated a significant correlation between the predictions made by the LR model and the observed MMP9 expression levels (*p* > 0.05, Fig. 4H). The DCA illustrated the clinical practicality of the LR model (Fig. 4I). Notably, patients with a high PS exhibited elevated MMP9 gene expression levels within the LR model framework (Fig. 4J). As illustrated in Fig. 4K and Fig. 4L, the mRMR method was used to select the top 20 features with the highest mRMR rankings. Subsequently, the RFE method was used to further refine the feature group selection process. Ultimately, six optimal features were identified for the SVM and LR modeling.

The AUC value and tenfold internal cross-validation of the SVM model were comparable to those of the LR model (Table 2). To validate ROC comparisons, we conducted the DeLong test, which revealed non-significant statistical differences between the two models (*p* = 0.956 and *p* = 0.698 in the training and validation sets, respectively). Based on these results, we used the PS generated by the SVM model for additional clinical analysis. To validate the prognostic significance of PS, Kaplan–Meier curves were employed, and an elevated PS was significantly associated with poor OS (*p* < 0.001, Fig. 4M). The median survival time was 10.76 and 14.93 months in the high- and low-PS groups, respectively.

**Table 2:** SVM and LR model performance.

	Training cohort					Validation cohort				
	AUC (95% CI)	ACC	SPE	SEN	*p*	AUC (95% CI)	ACC	SPE	SEN	*p*
SVM	0.778 (0.677–0.879)	0.731	0.653	0.862		0.754 (0.644–0.865)	0.731	0.673	0.828	
LR	0.776 (0.665–0.886)	0.744	0.735	0.759		0.722 (0.604–0.841)	0.731	0.776	0.655	
SVM vs. LR					0.957					0.698

*AUC* area under the curve, *SVM* support vector machine, *LR*, logistic regression, *ACC* accuracy, *SPE* specificity, *SEN* sensitivity.

### Biological mechanism and gene enrichment analysis of pathomics score

**Figure 5:**
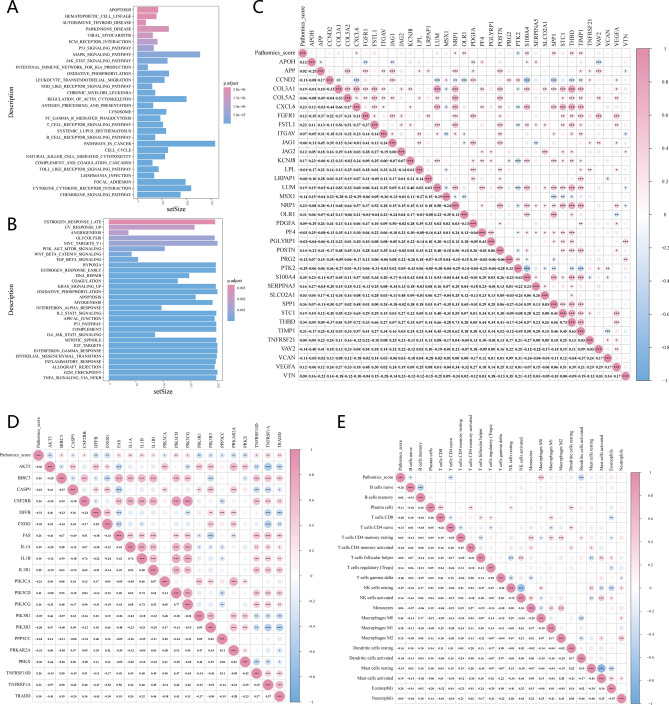
The biological mechanism and gene enrichment analysis of PS. (**A**) Gene set enrichment analysis of the KEGG and (**B**) Hallmark gene sets. (**C**) The correlation between PS and genes related to angiogenesis and (**D**) apoptosis. (**E**) Relationship between PS and immune infiltration.

To investigate the potential biological mechanism of PS, we first used the GSEA approach. We then analyzed the association between PS and immune infiltration in the tumor microenvironment and angiogenesis- and apoptosis-related genes. As shown in Fig. 5A**,** KEGG GSEA enrichment analysis revealed the top 30 pathways, among which the JAK-STAT, p53, and MAPK signaling pathways exhibited significant differentially expressed gene enrichment. As shown in Fig. 5B, GSEA enrichment analysis of the hallmark gene set revealed the top 30 pathways in which angiogenesis and apoptosis were significantly enriched. In Fig. 5C, the correlation between PS and angiogenesis-related gene expression was examined. PS showed a significant positive correlation with angiogenesis-related genes, including OLR1, S100A4, SPP1, THBD, and TIMP1, with *p* values < 0.01. In Fig. 5D, the correlation between PS and apoptosis-related gene expression was examined. PS exhibited a significant negative correlation with apoptosis-related genes, including AKT3, DFFB, EXOG, PIK3R1, and PRKAR2A, with *p* values < 0.05. As shown in Fig. 5E, variations in the fractions of the 22 immune cell types between patients with high and low PS were investigated. PS exhibited a significantly negative correlation with the infiltration of naïve CD4 + T cells and activated dendritic cells (*p* < 0.05).

## Discussion

We extracted HE-stained WSI features of GBM and used machine learning algorithms to predict MMP9 expression levels in patients. This paper presents the initial report of such findings for GBM based on the available information. These findings indicate that MMP9 expression is an independent prognostic factor for patients with GBM (HR = 1.917, 95% CI: 1.211–3.033). Among all extracted features, we constructed a classifier of the six most informative features that enabled the unambiguous classification of GBM patients with high MMP expression from those with low MMP9 expression (AUC = 0.754 in the test set). Furthermore, in the GSEA, the SVM model-derived PS exhibited significant correlations with angiogenesis, apoptosis, and tumor-related pathways, including the JAK-STAT, P53, and MAPK signaling pathways. Notably, PS was significantly associated with OS in patients with GBM (*p* = 0.002).

Previous investigations have demonstrated that MMP9 overexpression is correlated with poor prognosis and therapeutic outcomes in various types of malignancies, including colorectal^19^, renal^20^, and breast cancers^21^. The association between high MMP9 expression and poor prognosis was also demonstrated in patients with glioma^22^, while individuals with low-MMP9 expression experienced a notable increase in OS by 5.2 months when treated with bevacizumab^23^. This is consistent with the findings of our research. Our research demonstrated that patients exhibiting high levels of MMP9 expression had a poor prognosis, as evidenced by Kaplan–Meier curve analysis. Furthermore, multivariate analysis identified MMP9 as an independent risk factor for OS in patients with GBM.

GBM exhibits significant heterogeneity, and prognostic insights derived from key molecular marker expressions are valuable for clinical decision-making. Pathological examination plays a critical role in diagnosing and staging patients with GBM; however, its accuracy may be influenced by the expertise of the pathologists. Obtaining sufficient information from subjective assessments of tissue slices poses both challenges and opportunities. Recently, computer systems and medical image processing algorithms have been developed to assist in extracting image features related to tumor characteristics and survival outcomes^24,25^. Automated methods also offer the advantages of enhanced efficiency and reduced labor^26^.

Changes in tissue architecture and nuclear morphology often reflect molecular expression variations within tumors, which can be forecasted by deep learning algorithms from pathological images in breast^27^, prostate^28^, and gastrointestinal cancers^26^. In lung cancer, specific genetic mutations, such as STK11, EGFR, FAT1, SETBP1, KRAS, and TP53, have been predicted with an AUC range of 0.733–0.856, which was externally validated^29^. Moreover, Hollon et al.^30^ predict the molecular alterations (IDH mutation, 1p19q codeletion, and ATRX mutation) of diffuse gliomas using stimulated Raman histology and genomic data. Here, pathological image features demonstrated a good capability to categorize high and low MMP9 expression in patients with GBM. The two pathomics models exhibited strong predictive performance in both the training and validation subsets, with AUC values of 0.778 and 0.754, respectively, for the SVM model and 0.776 and 0.722, respectively, for the LR model. We inferred that pathomics was effective for predicting MMP9 expression.

We conducted additional investigations to examine the relationship between PS and OS in a pathomics cohort, using paired WSI and clinical data. Pathological image integration with deep learning has been used to predict the prognosis of several types of cancers^31–34^. Mobadersany et al. demonstrated a level of accuracy that exceeds the existing clinical framework in predicting patient outcomes for individuals diagnosed with glioma^35^. Another glioma study classified the survival rate of patients into four classes based on pathological images^36^. In this study, a high PS was significantly linked to a poor prognosis in GBM, which corroborated previous work. Taken together, our pathomics model shows considerable potential for risk stratification based on OS.

The correlation among pathology, imaging, and molecular and genetic data holds significant value in precision oncology. Given the high heterogeneity of tumors, the quantitative depiction of pathological and imaging phenotypes can more effectively compensate for microscopic molecular or genetic deficiencies. A correlation between pathological image information and gene expression in patients has been observed in several studies^37,38^. In this study, functional annotation was conducted using GSEA to uncover the underlying biological processes associated with PS. In the KEGG and Hallmark gene sets, we observed significant enrichment in apoptosis, angiogenesis, and signaling pathways implicated in cancer development, including the P53, JAK-STAT, and MAPK signaling pathways. There is strong evidence in the literature that these signaling pathways and phenotypes are intricately associated with GBM initiation and progression. The tumor P53 pathway is a classic therapeutic target with a complex regulatory network, the deregulation of which mediates GBM cell invasion, migration, and proliferation^39^. The JAK-STAT pathway is a key oncogenic hub in the GBM microenvironment, including reactive astrocytes, glioma, and immune cells, that impels invasion of tumor growth and treatment resistance^40^. The MAPK signaling pathway exhibits responsiveness to various stresses and plays an essential role in multiple cellular processes, including apoptosis. Alterations in MAPK signaling promote a malignant phenotype^41^. The findings may offer valuable knowledge regarding the molecular mechanisms underlying the morphological characteristics of GBM.

This study has several limitations. First, the study design was retrospective and inherently introduced potential confounding factors. Additionally, the study was conducted at a single center with a comparatively small sample size, necessitating further validation through large-scale multicenter studies. Third, patients included in this study were sourced from public datasets, which may have led to data heterogeneity. In future investigations, we intend to collect data for verification purposes.

In conclusion, MMP9 expression was significantly associated with the prognosis of patients with GBM. Prediction models based on the features of H&E-stained WSIs exhibit excellent stability and diagnostic efficiency, presenting a promising tool for predicting MMP9 expression in patients with GBM. With the advent of the big data era, pathomics integrated with genomics and proteomics has emerged as a new research direction for meeting the demand for precision medicine.

## Data Availability

The data presented in this study are available upon reasonable request. The related original data is publicly available and can be downloaded from TCIA (https://dev.cancerimagingarchive.net/) and TCGA (https://www.cancer.gov/ccg/research/genome-sequencing/tcga).

## Abbreviations

GBM: Glioblastoma
MMP9: Matrix metalloproteinase 9
SVM: Support vector machines
LR: Logistic regression
GSEA: Gene set enrichment analysis
ROC: Receiver operating characteristic
AUC: Area under the curve
OS: Overall survival
PS: Pathomics score
